# Long-term outcomes of tacrolimus conversion to sirolimus in kidney transplant recipients: an eight-year follow-up

**DOI:** 10.3389/fneph.2026.1813146

**Published:** 2026-05-25

**Authors:** Ibrahim Tawhari, Sook Park, Fatmah Yamani, Bing Ho, Mohammed Javeed Ansari, Mohammed Tawhari, Joseph Leventhal, Lorenzo Gallon

**Affiliations:** 1 Department of Internal Medicine, Nephrology, King Khalid University College of Medicine, Abha, Saudi Arabia; 2 Department of Medicine, Division of Nephrology, Northwestern University Feinberg School of Medicine, Chicago, IL, United States; 3 Department of Nephrology, King Faisal Specialist Hospital & Research Centre - Jeddah, Jeddah, Saudi Arabia; 4 College of Medicine, King Saud bin Abdulaziz University for Health Sciences, Riyadh, Saudi Arabia; 5 Department of Medicine, Division of Nephrology, King Abdulaziz Medical City in Riyadh, Ministry of National Guard – Health Affairs, Riyadh, Saudi Arabia; 6 King Abdullah International Medical Research Center, Riyadh, Saudi Arabia; 7 Department of Surgery, Comprehensive Transplant Center, Northwestern University Feinberg School of Medicine, Chicago, IL, United States; 8 University of Illinois Chicago, Chicago, IL, United States

**Keywords:** calcineurin inhibitors, conversion, kidney transplantation, long-term, outcomes, sirolimus

## Abstract

**Objective:**

Calcineurin inhibitors, such as tacrolimus, are associated with long-term nephrotoxicity and renal allograft dysfunction despite evidence of improved kidney transplantation outcomes. Consequently, minimizing calcineurin inhibitor exposure has attracted considerable interest. This study aimed to investigate the long-term outcomes of late conversion of tacrolimus to sirolimus in a steroid-free immunosuppressive regimen in kidney transplant recipients.

**Methods:**

We conducted an 8-year retrospective analysis of outcomes in 207 kidney transplant recipients enrolled in a previous randomized clinical trial, in which patients receiving maintenance therapy with tacrolimus and mycophenolate were randomized 2:1 at 6–12 months post-transplantation to conversion from tacrolimus to sirolimus or continuation of tacrolimus.

**Results:**

Over the 8-year post-transplant follow-up, the sirolimus cohort exhibited a superior and more preserved GFR compared to the tacrolimus cohort, with a total GFR change of −1.18 mL/min/1.73 m² (95% CI, −4.86 to 3.06) with sirolimus versus −9.57 mL/min/1.73 m² (95% CI, −16.92 to −7.26) with tacrolimus, with a between-group difference of −8.39 mL/min/1.73 m². The two groups did not significantly differ in terms of graft loss, patient survival, or *de novo* donor-specific antibody development. The sirolimus group showed a numerically but insignificantly elevated biopsy-proven acute rejection rate. Adverse effect profiles revealed that the sirolimus group was associated with significantly lower BK viremia and cancer rates, whereas other adverse events did not significantly differ between the groups.

**Conclusions:**

Delayed conversion from tacrolimus to sirolimus (≥6 months post-transplantation) was associated with long-term preserved estimated glomerular filtration rate without a significantly increased risk of renal allograft rejection or graft loss.

## 1 Introduction

For over three decades, calcineurin inhibitors (CNIs), such as tacrolimus (TAC), have improved the long-term outcomes of organ transplantation, particularly kidney transplantation. However, these drugs are nephrotoxic, and solid-organ transplantation is frequently associated with renal insufficiency (1). In addition to nephrotoxicity, cardiovascular risk factors, including hypertension, dyslipidemia, and glucose intolerance, have been associated with CNIs (2). In surviving kidney transplant recipients, interstitial fibrosis and tubular atrophy are the major causes of late-stage graft loss. Long-term CNI use is associated with progressive renal function deterioration, as observed in renal biopsies revealing interstitial fibrosis, tubular atrophy, vascular occlusive changes, and glomerulosclerosis (3, 4). Various approaches reduce or eliminate CNI exposure, including (1) CNI minimization, which reduces drug exposure; (2) CNI conversion, which decreases CNI dosage with an alternative agent until complete replacement; (3) CNI withdrawal, which is gradually tapering the medication dose until elimination; and (4) CNI avoidance, which replaces the CNI with another immunosuppressive treatment (5). The use of mammalian targets of rapamycin inhibitors (mTORis), including sirolimus (SRL) and everolimus, as substitutes to CNI use in kidney transplantation has gained significant interest in the recent decade (6, 7). However, randomized controlled trials (RCTs) have revealed that the *de novo* use of low- or high-dose mTORi during kidney transplantation for replacing CNIs has been associated with high rejection rates, graft failure, and poor tolerability (6–12). Subsequently, several RCTs have evaluated the impacts of delaying the introduction of mTORi. A systematic review by Lim et al., encompassing 29 RCTs, revealed that delayed conversion of CNI to mTORi was associated with short-term improvements in estimated glomerular filtration rate (eGFR) at 1 year post-transplantation (13). Nevertheless, studies evaluating the long-term outcomes of this treatment strategy are scarce. A long-term follow-up of kidney transplant recipients in the Spare-the-Nephron trial by Weir et al. reported an association between early conversion from CNI/mycophenolate acid (MPA) to SRL/MPA at ≤6 months post-transplantation and improved eGFR at 1 year post-transplantation; however, GFRs did not significantly differ at 2- and 8-year follow-ups. In that study, most patients received corticosteroids (89.8%) alongside CNI/MPA or SRL/MPA (14). Therefore, this study aimed to evaluate the long-term outcomes of late conversion of CNI to SRL using a steroid-free immunosuppressive regimen in kidney transplant recipients.

## 2 Materials and methods

### 2.1 Participants

**Figure 1:**
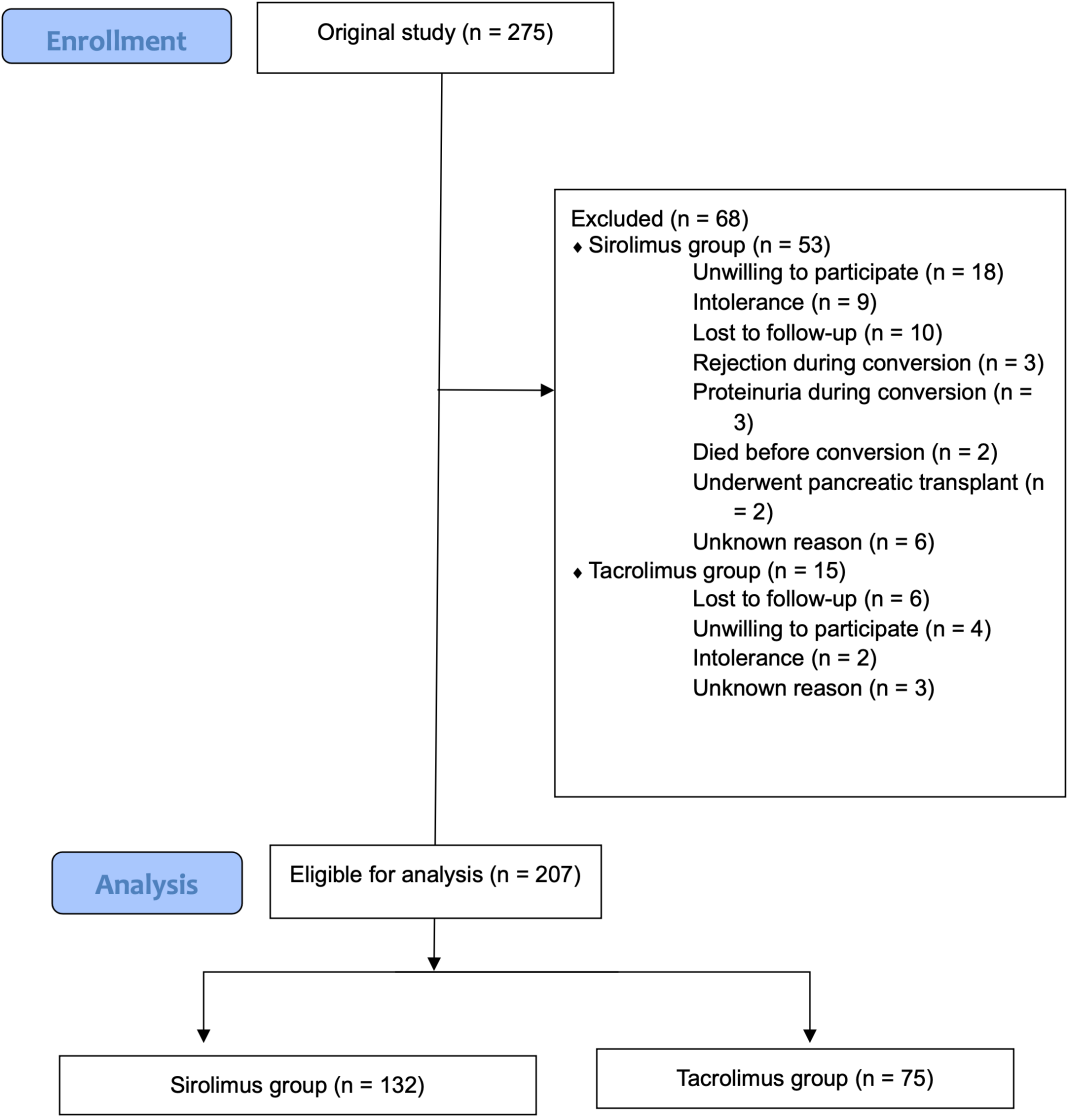
CONSORT diagram. The diagram illustrates the participant flow throughout the study. Overall, 207 participants are randomized into two groups, including 132 and 75 in the sirolimus and tacrolimus groups, respectively. Outcomes are analyzed and compared between both groups. The intention-to-treat analysis approach is employed.

The original study was a prospective open-label single-center randomized trial (clinical trial number: NCT00866879) that was initiated in 2007 and enrolled 275 patients. The participants were randomized (2:1) to undergo conversion from TAC to SRL or continue TAC between 6 and 12 months post-transplantation. Subsequently, participants were followed up for 24 months following randomization, as previously reported (15). However, 68 of the initial 180 patients (37.38%) and 15 of the initial 90 patients (16.67%) in the SRL and TAC groups, respectively, were excluded during that original study for various reasons (**Figure 1**). Most of those who withdrew from the SRL group initially provided consent to enroll but later decided not to participate. Both groups had comparable baseline characteristics despite an unequal exclusion rate. All participants received induction immunosuppressive therapy with alemtuzumab and underwent early steroid withdrawal. Data from participants who completed the first part of this study were retrospectively collected for up to 8 years. The Institutional Review Board of Northwestern University approved this study (IRB # STU00209482).

Kidney transplant recipients aged 18–70 years were screened for the initial study. The following were the exclusion criteria: kidney transplant recipients with severe proteinuria during randomization (>2 g/day), eGFR by the Modification of Diet in Renal Disease (MDRD) equation of <40 mL/min/1.73 m^2^, history of >2 episodes of acute cellular rejection (ACR) or >Banff 1 ACR within 3 months prerandomization, history of primary focal segmental glomerulosclerosis, multi-organ transplant, significant or active infection (e.g., human immunodeficiency virus, hepatitis B virus or hepatitis C virus, severe hyperlipidemia uncontrolled with statins, platelet count of <100,000 mm^3^, or white blood cell count of <2,000 mm^3^), pregnant or nursing, and history of malignancy post-transplantation (except for treated basal or squamous cell skin cancer).

All biopsy-proven acute rejection episodes were graded according to the current Banff classification at the time of biopsy. ACR episodes were managed on the basis of severity using pulsed methylprednisolone, followed by an oral prednisone taper and rabbit anti-thymocyte globulin. Intravenous pulsed methylprednisolone, therapeutic plasma exchange, intravenous immunoglobulin, and rituximab were employed for treating antibody-mediated rejection (AMR) episodes.

### 2.2 Outcomes

The change in the eGFR according to the MDRD at 8 years was the primary endpoint. Graft loss (e.g., death-censored graft loss, return to dialysis, and re-transplantation), incidence of biopsy-proven acute rejection (e.g., cellular and AMR), and incidence of *de novo* donor-specific antibody (DSA) development were the secondary endpoints.

### 2.3 Data

Data on major adverse events, including infection, malignancy, posttransplant diabetes, hyperlipidemia, proteinuria, neutropenia, edema, hair loss, oral ulcers, and tremors, were collected. Data on eGFR, urine dipstick protein (classified as binary, with ≥ +1 as a positive), graft failure, kidney biopsies, and patient death status were directly retrieved from the Northwestern Medicine Enterprise Data Warehouse. Moreover, DSA and *de novo* DSA data were collected. As urine protein/creatinine and microalbumin/creatinine ratios were not routinely collected, urine dipstick protein was used as a marker for proteinuria.

### 2.4 Statistical analysis

We used descriptive statistics for summarizing the data, including means and standard deviations (SDs) for continuous variables and frequencies and counts for categorical variables. Baseline patient characteristics were compared between the TAC and SRL groups using Fisher’s exact test and two-sample t-tests or Mann–Whitney U-tests for categorical and continuous variables, respectively, depending on the normality of data distribution. To determine patient survival, graft survival, acute rejection, and adverse event occurrence, Kaplan–Meier survival estimates were employed. Cumulative curves were compared between the TAC and SRL groups using the log-rank and cox proportional hazard tests. A mixed-effect model was used for comparing eGFR slopes over time between the two groups. P-values were calculated using a two-tailed test; a p-value of <0.05 was considered statistically significant.

## 3 Results

### 3.1 Participants

A total of 207 kidney transplant recipients were included in this retrospective analysis, of whom 132 and 75 were randomized to the SRL and TAC groups, respectively (**Figure 1**). The participants’ baseline characteristics are summarized in **Table 1**. At enrollment, both groups did not vary in age, sex, race, primary cause of kidney disease, donor type, sensitization, and delayed graft function incidence.

**Table 1:** Baseline characteristics.

Characteristic	Tacrolimus	Sirolimus
Number of patients	75	132
Age at KT (mean [SD])	49.36 (12.70)	48.59 (11.64)
Male (%)	48 (64.0)	75 (56.8)
Race (%)		
White	37 (49.3)	63 (47.7)
African–American	21 (28.0)	24 (18.2)
Other	17 (22.7)	45 (34.1)
Primary ESRD cause (%)		
DM	16 (21.3)	42 (31.8)
GN	20 (26.7)	39 (29.5)
HTN	23 (30.7)	28 (21.2)
PCKD	9 (12.0)	12 (9.1)
Obstructive uropathy	0 (0.0)	5 (3.8)
Chronic pyelonephritis	2 (2.7)	2 (1.5)
Congenital renal agenesis	3 (4.0)	3 (2.3)
Other	2 (2.7)	1 (0.8)
Donor type (%)		
Deceased	23 (30.7)	43 (32.6)
Living related	26 (34.7)	49 (37.1)
Living unrelated	26 (34.7)	40 (30.3)
HLA mismatch (mean [SD])	3.88 (1.63)	3.79 (1.77)
Positive PRA (%)	14 (23.3)	24 (23.8)
DGF (%)	7 (9.6)	13 (10.1)

KT, kidney transplant; SD, standard deviation; ESRD, end-stage renal disease; DM, diabetes mellitus; GN, glomerulonephritis; HTN, hypertension; PCKD, polycystic kidney disease; HLA, human leukocyte antigen; PRA, panel reactive antibody; DGF, delayed graft function.

Tx_date, transplant date; NODAT, posttransplant diabetes mellitus; BKV, BK polyoma viremia; BCC, basal cell carcinoma; Colon CA, colon cancer; Lung CA, lung cancer; Ovarian CA, ovarian cancer; PTLD, posttransplant lymphoproliferative disorder; C. diff, Clostridium difficile; CMV, cytomegalovirus; URI, upper respiratory tract infection; UTI, urinary tract infection.

Notably, at specific time points, data were available from 96.6%, 79.2%, and 62.32% of the included participants at 72, 84, and 96 months, respectively.

Over the follow-up period, the SRL level was maintained at 6–8 ng/mL, and the TAC level was maintained at 5–7 ng/mL.

### 3.2 Renal allograft function

**Figure 2:**
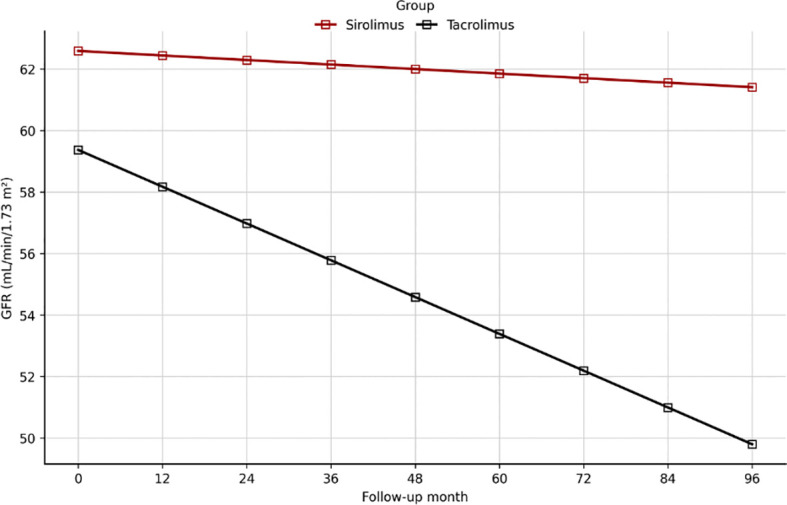
Effect of treatment on the estimated glomerular filtration rate (eGFR). This graph depicts the change in GFR over 96 months post transplantation for the sirolimus and tacrolimus groups, from the adjusted mixed-effects model demonstrating an a relatively preserved GFR with sirolimus and a significantly steeper decline with tacrolimus.

A mixed-effect model analysis, adjusted for baseline GFR, HLA antigen mismatch, donor type (living versus deceased), delayed graft function, diabetes mellitus, hypertension, infectious complications, and the number of rejection episodes, revealed a more preserved GFR with SRL. Over the 8-year post-transplant period, the SRL group showed a total change in GFR by −1.18 mL/min/1.73 m² (95% CI, −4.86 to 3.06) from the baseline, compared to −9.57 mL/min/1.73 m² (95% CI, −16.92 to −7.26) in the TAC group, with a between-group difference of −8.39 mL/min/1.73 m². The estimated annual change in GFR was −0.15 mL/min/1.73 m²/year (95% CI, −0.81 to 0.51) with SRL group compared to −1.20 mL/min/1.73 m²/year (95% CI, −2.12 to −0.91) with TAC, with an annual between-group difference of −1.05 mL/min/1.73 m²/year (**Figure 2**).

The mean eGFR was assessed at various time points during an 8-year follow-up study where SRL and TAC treatments were compared. As summarized in **Table 2**, the mean eGFRs between the two groups did not significantly differ at baseline (SRL, 62.59 [SD, 16.47] mL/min/1.73 m^2^; TAC, 59.37 [SD, 13.49] mL/min/1.73 m^2^; p = 0.170) and 24 months post-transplantation (SRL, 69.66 [SD, 20.36] mL/min/1.73 m^2^; TAC, 65.08 [SD, 18.97] mL/min/1.73 m^2^; p = 0.130). At 36 months post-transplantation, the difference became statistically significant (SRL, 68.49 [SD, 18.65] mL/min/1.73 m^2^; TAC, 60.39 [SD, 14.97] mL/min/1.73 m^2^; p = 0.130). This trend continued throughout the 96-month follow-up period.

**Table 2:** Mean eGFR of the study groups at different time points.

eGFR (mL/min/1.73 m^2^) Mean (SD)			
Time post-transplantation	Sirolimus	Tacrolimus	p-value
Baseline	62.59 (16.47)	59.37 (13.49)	0.170
24 months	69.66 (20.36)	65.08 (18.97)	0.130
36 months	68.40 (18.31)	59.14 (16.78)	0.010
48 months	66.49 (18.65)	60.39 (14.97)	0.016
60 months	67.78 (18.19)	61.48 (15.84)	0.015
72 months	67.31 (14.71)	56.69 (16.69)	<0.001
84 months	69.00 (14.50)	52.94 (12.33)	<0.001
96 months	67.34 (11.06)	53.74 (9.12)	<0.001

eGFR, estimated glomerular filtration rate.

At 96 months, the mean eGFRs were 67.34 (SD, 11.06) and 53.74 (SD, 9.12) mL/min/1.73 m^2^ in the SRL and TAC groups, respectively (p < 0.001) (**Table 2**).

### 3.3 Patient and renal allograft survival

**Figure 3:**
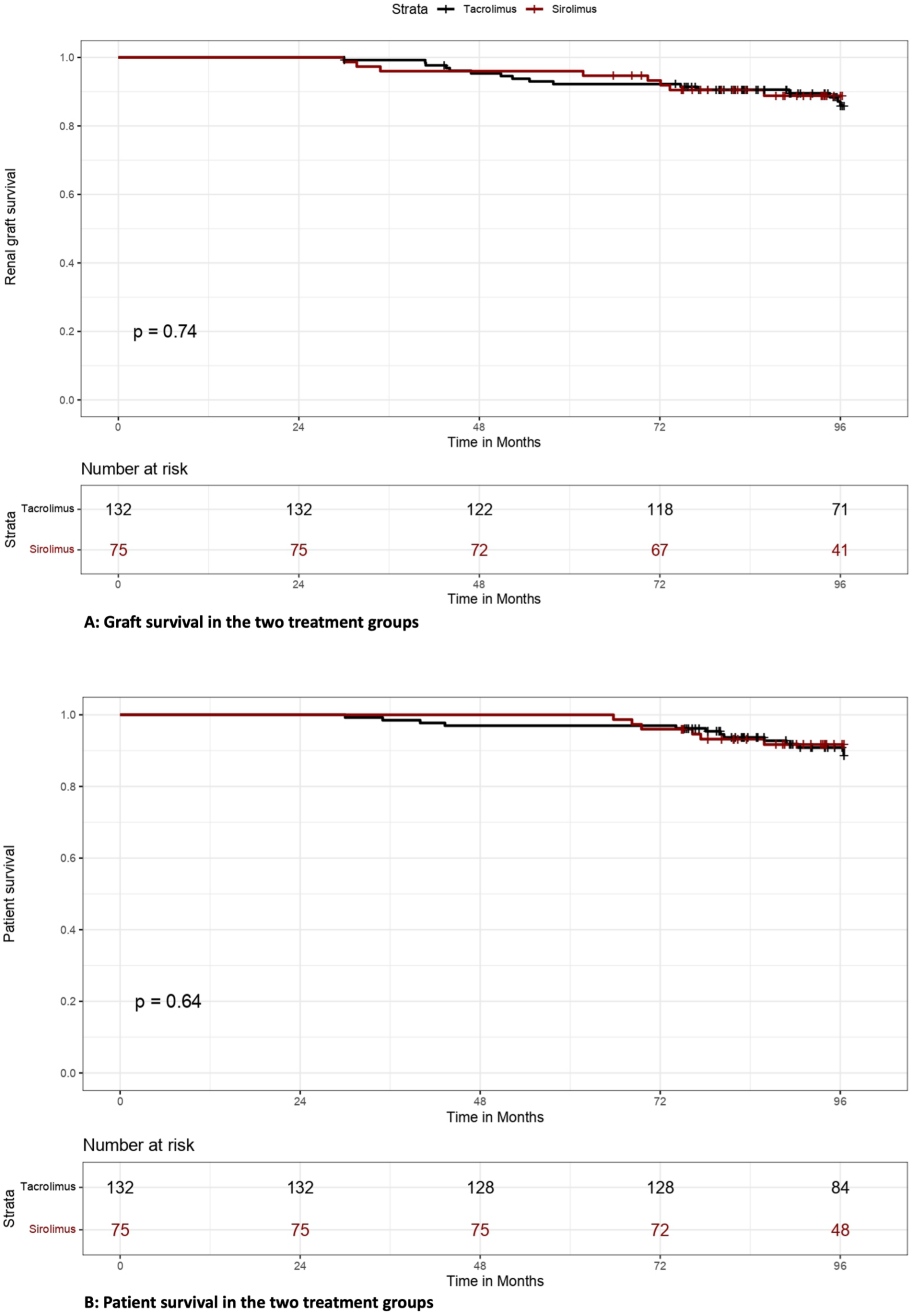
Effect of tacrolimus to sirolimus conversion on graft and patient survival. The Kaplan–Meier survival rates are not significantly different between the two groups for both renal graft survival **(A)** and patient survival **(B)**.

Kaplan–Meier graft and patient survival rates did not significantly differ between the two groups (**Figures 3A, B**). The Cox proportional hazard ratio (HR) for comparing SRL and TAC was 1.15 (95% CI, 0.49–2.70) for time to graft loss and 1.26 (95% CI, 0.47–3.31) for time to death at 8 years post-transplantation.

### 3.4 Biopsy-proven acute rejection incidence

The Kaplan–Meier cumulative incidences of acute rejection in the SRL and TAC groups were 1.5% and 1.3%, 8.3% and 4%, 10.6% and 5.3%, and 10.6% and 6.7% at 12, 48, 72, and 96 months, respectively. Most of these biopsies (70%) were for cause, and the remaining 30% were protocol biopsies. Further analysis of the types of acute rejection episodes revealed that ACR developed in 5.3% of patients in both the SRL and TAC groups, with no statistically significant differences noted between them (p = 1.000). AMR was observed in 4.5% and 1.4% of patients in the SRL and TAC groups, respectively; however, the difference was not significant (p = 0.219). Few cases developed mixed cellular and AMR rejection, including 0.8% and 1.3% in the SRL and TAC groups, respectively; however, the difference was not statistically significant (p = 0.684).

Furthermore, the ACR according to Banff classification subtypes within each treatment group occurred as follows: Banff IA in 3.03% and 2.67% of the SRL and TAC groups, respectively, without significant differences (p = 0.881); Banff IB in 1.52% and 1.33% of the SRL and TAC groups, respectively, without a statistically significant difference (p = 0.916); Banff IIA rejections in 0.76% of the SRL group, whereas none were observed in the TAC group (p = 0.691); the treatment groups had no instances of Banff IIB, III, or IV subtype rejections.

**Figure 4:**
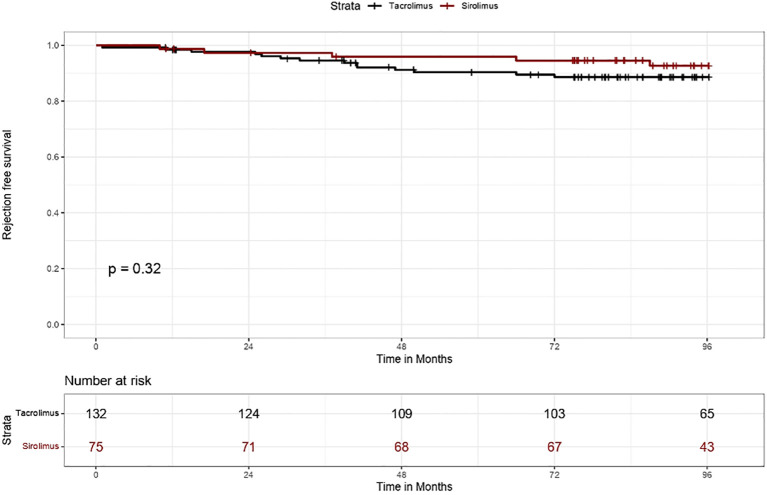
Effect of treatment on biopsy-proven acute rejection. The Kaplan–Meier curve depicting the time to occurrence of biopsy-proven acute rejection in the two treatment groups showing no significant difference in acute rejection between both groups.

At 96 months post-transplantation, the HR for comparing acute rejection between SRL and TAC treatments was 1.68 (95% CI, 0.60–4.66) (**Figure 4**).

### 3.5 Donor-specific antibodies

At 96 months, the cumulative event rates of the development of *de novo* DSA class I for SRL and TAC were 8.33% and 10.67%, respectively (p = 0.758), whereas those of DSA class II were 9.09% and 12% for SRL and TAC, respectively (p = 0.669).

### 3.6 Adverse events

Cox proportional hazards models revealed that SRL was associated with a significantly lower hazard of malignancy (adjusted HR, 0.24; 95% CI, 0.06–0.96) and BK viremia (adjusted HR, 0.47; 95% CI, 0.24–0.94) but a significantly increased hazard of neutropenia (adjusted HR, 1.59; 95% CI, 1.23–1.98). No statistically significant differences were noted between the two groups for proteinuria (adjusted HR, 2.21; 95% CI, 0.98–3.53), infection (adjusted HR, 1.52; 95% CI, 0.83–2.80), cytomegalovirus (CMV) viremia (adjusted HR, 0.36; 95% CI, 0.07–2.00), posttransplant diabetes (adjusted HR, 3.52; 95% CI, 0.41–30.34), or hyperlipidemia (adjusted HR, 1.07; 95% CI, 0.35–3.30) (**Table 3**).

**Table 3:** Hazard ratios for adverse events comparing sirolimus vs tacrolimus.

Outcome	Adjusted HR (95% CI)
Malignancy	0.24 (0.06–0.96)
Any infection	1.52 (0.83–2.80)
CMV viremia	0.36 (0.07–2.00)
Neutropenia	1.59 (1.23–1.98)
Post-transplant diabetes	3.52 (0.41–30.34)
Hypertriglyceridemia	1.07 (0.35–3.30)
Proteinuria	2.21 (0.98–3.53)
BK viremia	0.47 (0.24–0.94)

CMV, cytomegalovirus.

## 4 Discussion

The long-term use of CNIs has been linked to various adverse outcomes, including renal dysfunction. Previous RCTs have that mTORi used as a primary immunosuppressant in kidney transplants was associated with poor renal allograft outcomes (8–11). However, in 2006, a meta-analysis of 33 RCTs investigated mTORi use in kidney transplantation and revealed an association between mTORi treatment, with or without CNIs, and enhanced renal allograft function (7). Of the 33 RCTs included in that meta-analysis, 8 studies involving 750 participants explored mTORi use as a replacement for CNIs, indicating no differences in acute rejection or a higher eGFR associated with mTORi use for 2 years post-transplantation. Moreover, higher leukopenia, thrombocytopenia, and anemia rates were observed. Nevertheless, data on the long-term outcomes of replacing CNIs with mTORi beyond 2 years post-transplantation remain limited. Our study demonstrated that delayed conversion (> 6 months post-transplantation) from TAC to an SRL-based regimen in kidney transplant recipients was associated with a significantly higher long-term eGFR, which was sustained at 8 years post-transplantation. Although the difference in the mean eGFR did not show statistical significance at 12 and 24 months, as reported in the previous publication of this study, it became significant at 36 months and was maintained throughout the 96-month follow-up period. This finding suggests that chronic CNI-induced nephrotoxicity may require prolonged CNI exposure to develop. The adjusted mixed effects model further supported this finding, describing a more stable eGFR with SRL than a steeper decline with TAC in the long term. Differences between crude GFR means and model-adjusted estimates are expected, as the model incorporates all available repeated measurements and adjusts for relevant clinical covariates that may influence the mean eGFR at different time points. Our observation aligns with those of a previous longitudinal histological study, demonstrating that CNI-induced nephrotoxicity is nearly present in all allografts at 10 years post-transplantation (16).

The graft and patient survival rates were comparable between the two groups; however, the incidence of biopsy-proven acute rejection was higher in the SRL group, although the difference was statistically insignificant. Our results align with those noted in previous studies, including the CONVERT and CONCEPT trials (17, 18). Furthermore, a meta-analysis of 29 open-label RCTs conducted in 2012 by Lim et al. support our findings; however, data beyond 2 years post-randomization were unavailable for most of the RCTs included in the analysis (13). An 8-year follow-up of kidney transplant recipients enrolled in the Spare-the-Nephron trial revealed that compared with the CNI group, the SRL group exhibited a trend toward improved long-term renal function. However, only a few participants completed the 8-year follow-up; therefore, the results were not statistically significant. Similarly, the SRL group showed a numerically (but not statistically significant) higher acute rejection rate and no increased risk of graft loss (14).

The development of proteinuria following conversion from CNI to mTORi has been reported and is frequently an indicator of poor renal outcomes (19). Our study revealed a numerically higher hazard of new-onset proteinuria with SRL conversion to SRL, but the difference was not statistically significant. However, the number of patients with complete spot urine protein-to-creatinine ratios was limited, hindering the generalization of this observation. In addition, the development of proteinuria in patients receiving SRL may be dose-dependent and often associated with SRL levels of >10 ng/mL (20). We herein maintained 6–8-ng/mL SRL levels, potentially influencing the incidence or severity of the observed proteinuria.

No association was observed between CNI-free SRL-based regimen with MPA and an elevated risk of new-onset diabetes, hyperlipidemia, or infectious events. However, an increased risk of neutropenia was noted with SRL use, aligning with the findings of previous RCTs (6–12). A CNI-free SRL regimen was associated with a significantly lower BK viremia incidence. A retrospective study has reported that a SRL-based regimen led to a reduced BK viremia incidence (21); however, this approach is infrequently employed and has not been explored in prospective RCTs. It is unclear whether this can be attributed to the lower-intensity immunosuppressive property of SRL or a specific effect of SRL on the BK virus. SRL may have a lower inhibitory effect on BK-specific cytotoxic T cells than TAC, as it inhibits IL-2-dependent proliferation of T cells and not the release of IL-2 by lymphocytes, as does TAC (22, 23). Conversely, SRL may exhibit anti-BK viral activity, as shown in an *in vitro* study (24). Similar to observations in previous studies, our study showed a significantly lower incidence rate of malignancies in patients converted to SRL (24), possibly owing to its antineoplastic properties (25). Similarly, a decreased risk of CMV viremia may be associated with SRL conversion. This finding is consistent with the results of several studies that demonstrated a reduced incidence of CMV viremia in the SRL-based regimen compared with the TAC-based regimen (9, 26–29).

Our study is one of the largest long-term follow-up studies that evaluated the outcomes of delayed conversion of TAC to SRL in kidney transplant recipients with follow-up extended up to 8 years. However, our study had some limitations that could impact the interpretation and robustness of our findings. The retrospective nature of our study and the lack of standardized data collection intervals could influence the accuracy and reliability of our results. Although we accounted for some factors that may affect the GFR in the mixed-effects model, it was not feasible to adjust for other potential confounders such as the age of the donor, donor comorbidities, longitudinal blood pressure and glycemic control, severity of proteinuria, severity of rejection, adherence to medication, and trough-level variability of tacrolimus or sirolimus, as these factors were not consistently available over time across the extended follow-up. The inability to assess medication adherence and potential variation in immunosuppressant trough levels may have also introduced a confounding effect on other endpoints such as viremia, allograft rejection, graft survival, and the development of *de novo* DSA. In addition, the marked decline in the patient population after year 6 may raise a concern for potential attrition bias. Despite these limitations, several participants had complete data for prospectively defined outcomes at the 6-year mark (9s6.6%) and 7-year follow-up (approximately 80%). Notably, we consistently observed a higher eGFR among those receiving the SRL regimen. The loss of follow-up is frequent in studies with long follow-up durations as patients may die or relocate during the study (30). Lastly, our study exclusively enrolled patients with low immunological risk, likely limiting the generalizability of our findings to broader patient populations with varying immunological risks.

In conclusion, our study offers some of the longest available follow-up data on the efficacy and safety outcomes of SLR- and TAC-based immunosuppressive regimens with MPA in kidney transplant recipients. Our results revealed that delayed conversion to an SLR-based regimen was associated with long-term preserved eGFR without increasing the risk of acute rejection or graft loss in kidney transplant recipients. However, more studies are required to identify the characteristics of kidney transplant recipients who may benefit from switching from TAC to SRL and those that would have negative outcomes, such as allograft rejection and loss.

## Data Availability

The data analyzed in this study is subject to the following licenses/restrictions: De-identified data may be provided upon reasonable request from the corresponding author, subject to institutional approvals. Requests to access these datasets should be directed to Ibrahim Tawhari, ihtawhari@kku.edu.sa.
